# Pharmacogenomics of Interferon-ß Therapy in Multiple Sclerosis: Baseline IFN Signature Determines Pharmacological Differences between Patients

**DOI:** 10.1371/journal.pone.0001927

**Published:** 2008-04-02

**Authors:** Lisa G. M. van Baarsen, Saskia Vosslamber, Marianne Tijssen, Josefien M. C. Baggen, Laura F. van der Voort, Joep Killestein, Tineke C. T. M. van der Pouw Kraan, Chris H. Polman, Cornelis L. Verweij

**Affiliations:** 1 Department of Molecular Cell Biology and Immunology, VU Medical Center, Amsterdam, The Netherlands; 2 Department of Pathology, VU Medical Center, Amsterdam, The Netherlands; 3 Department of Neurology, VU Medical Center, Amsterdam, The Netherlands; University of Vienna, Austria

## Abstract

**Background:**

Multiple sclerosis (MS) is a heterogeneous disease. In order to understand the partial responsiveness to IFNß in Relapsing Remitting MS (RRMS) we studied the pharmacological effects of IFNß therapy.

**Methodology:**

Large scale gene expression profiling was performed on peripheral blood of 16 RRMS patients at baseline and one month after the start of IFNß therapy. Differential gene expression was analyzed by Significance Analysis of Microarrays. Subsequent expression analyses on specific genes were performed after three and six months of treatment. Peripheral blood mononuclear cells (PBMC) were isolated and stimulated *in vitro* with IFNß. Genes of interest were measured and validated by quantitative realtime PCR. An independent group of 30 RRMS patients was used for validation.

**Principal Findings:**

Pharmacogenomics revealed a marked variation in the pharmacological response to IFNß between patients. A total of 126 genes were upregulated in a subset of patients whereas in other patients these genes were downregulated or unchanged after one month of IFNß therapy. Most interestingly, we observed that the extent of the pharmacological response correlates negatively with the baseline expression of a specific set of 15 IFN response genes (R = −0.7208; p = 0.0016). The negative correlation was maintained after three (R = −0.7363; p = 0.0027) and six (R = −0.8154; p = 0.0004) months of treatment, as determined by gene expression levels of the most significant correlating gene. Similar results were obtained in an independent group of patients (n = 30; R = −0.4719; p = 0.0085). Moreover, the *ex vivo* results could be confirmed by *in vitro* stimulation of purified PBMCs at baseline with IFNß indicating that differential responsiveness to IFNß is an intrinsic feature of peripheral blood cells at baseline.

**Conclusion:**

These data imply that the expression levels of IFN response genes in the peripheral blood of MS patients prior to treatment could serve a role as biomarker for the differential clinical response to IFNß.

## Introduction

Multiple sclerosis (MS) is a common inflammatory disease of the central nervous system characterized by progressive neurological dysfunction. The disease has a heterogeneous nature, which is reflected in the clinical presentation, ranging from mild to severe demyelinating disease. No curative therapy is currently available, and the majority of affected individuals are ultimately disabled.[1]

IFNs were the first agents to show clinical efficacy in RRMS. Interferon beta (IFNß) decreases clinical relapses, reduces brain disease activity, and possibly slows down progression of disability. However, therapy is associated with a number of adverse reactions, including flu-like symptoms and transient laboratory abnormalities. Moreover, the response to IFNß is partial, i.e. disease activity is suppressed by only about one third.[2] Clinical experience suggests that there are IFN ‘responders’ as well as ‘non responders’, however clear criteria for such classification are still lacking.[3] In the absence of predictive biomarkers the question remains who will respond to therapy and who to treat when inconvenience and costs are significant.

Part of the unresponsiveness to IFNß can be explained by immunogenicity. However, since not all unresponsive patients develop neutralizing antibodies (Nabs), and Nabs can disappear over time,[4]–[7] other mechanisms have to be involved to explain unresponsiveness. Hence, there have to be biological disease mechanisms in a subpopulation of patients that results in insensitivity or resistance to the effects of IFNs. This implies that pharmacological responses may differ between patients, leading to inter-individual differences in clinical efficacy. We hypothesize that an in depth understanding of the pharmacological factors underlying the therapeutic mechanisms and therapy unresponsiveness is the key for the identification of predictive markers.

In normal physiology type I IFNs achieve their biological effects by binding to multi-subunit receptors IFNAR-1 and -2 on the cell surface, thereby initiating a complex cascade of intracellular secondary messengers that emerge in two divergent pathways. One pathway, leads to activation of the transcription factor IFN-stimulated gene factor 3 (ISGF3), a complex of phosphorylated Signal Transducer and Activator of Transcription (STAT) 2 with STAT1 and IFN regulatory factor 9 (IRF-9; p48) that binds to the IFN-stimulated response element (ISRE) present in multiple genes.[8], [9] The other pathway involves STAT2/1 and STAT2/3 heterodimers and STAT1 homodimer (IFN-α-activated factor, AAF), which bind to the IFN gamma-activated sequence (GAS) response element.[9]–[12] Ultimately, the IFN-induced activation of ISRE and GAS enhancer elements switch on a wide variety of genes[13] leading to specific transcriptional changes.

With the aid of genomics technology, we are now in a position to provide sufficient knowledge to determine pharmacological outcomes that will allow us to search for predictors of therapeutic outcomes. Previously we demonstrated that gene expression signatures in MS may differ significantly between patients.[14] We found that a subgroup of MS was characterized by an increased expression of an immune defense response gene set, including a type I IFN response signature. Here we generated and analyzed pre- and post- IFNß treatment gene expression patterns of RRMS patients with the aim of identifying pre-existing and/or drug-induced signatures that will allow us to make predictions on the expected pharmacological effects of IFNß treatment. We show that the expression level of IFN response genes prior to treatment, could serve a role as biomarker for the pharmacological differences between patients with MS at the molecular level.

## Methods

### Patients

A first group of 16 Dutch patients (10 females and 6 males) and a second group of 30 Dutch patients (17 females and 13 males) with clinically definite relapsing-remitting MS was recruited from the outpatient clinic of the MS Centre Amsterdam. Mean age at start of IFNß therapy for the test group is 40.6±7.7, mean EDSS is 2.3±1.3 (range 1–6). Blood samples were obtained at a fixed time during the day just before treatment and 1, 3 and 6 months after start of the therapy. Patients received either Avonex (n = 4), Betaferon (n = 7), Rebif 22 ( = 2) or Rebif 44 (n = 3). For the validation group, mean age at start of IFNß therapy is 34.0±9.9, mean EDSS 2.3±1.1 (range 0–4.5). Patients received either Avonex (n = 7), Betaferon (n = 8), Rebif 22 (n = 4) or Rebif 44 (n = 11).

The study was approved by the ethics committee of the VUmc and all patients provided written informed consent.

### Blood sampling

From each patient blood was drawn into one PAXgene tube (PreAnalytix, GmbH, Germany) and three heparin tubes (Beckton Dickinson, Alphen a/d Rijn, Netherlands). After blood collection, tubes were transferred from the clinic to the lab within one hour in order to isolate fresh peripheral blood mononuclear cells (PBMCs) from heparinized blood using lymphoprep (Axis-Shield, Lucron) density gradient centrifugation. PAXgene tubes were stored at room temperature (RT) for two hours to ensure complete lyses of all blood cells after which tubes were stored at −20 until RNA isolation. Total RNA was isolated within 7 months after storage. Tubes were thawed 2 hours at RT prior to RNA isolation. Next, RNA was isolated using the PreAnalytix RNA isolation kit according to the manufacturers' instructions, including a DNAse (Qiagen, Venlo, Netherlands) step to remove genomic DNA. Quantity and purity of the RNA was tested using the Nanodrop spectrophotometer (Nanodrop Technologies, Wilmington, Delaware USA).

### Microarray hybridization

We used 43K cDNA microarrays from the Stanford Functional Genomics Facility (http://microarray.org/sfgf/) printed on aminosilane-coated slides containing ∼20.000 unique genes. First DNA spots were UV-crosslinked to the slide using 150–300 mJoules. Prior to sample hybridisation, slides were prehybridized at 42 degrees Celsius for 15 minutes in a solution containing 40% ultra pure formamide (Invitrogen, Breda, Netherlands), 5% SSC (Biochemika, Sigma), 0.1% SDS (Fluka Chemie, GmbH, Switserland) and 50 µg/ml BSA (Panvera, Madison, USA). After prehybridization slides were briefly rinsed in MilliQ water, thoroughly washed in boiling water and 95% ethanol and air-dried. Sample preparation and microarray hybridisation was performed as described previously,[15] apart from the different postprocessing and prehybridization described above.

### Microarray analysis

Data storage and filtering was performed using the Stanford Microarray Database[16] (http://genome-www5.stanford.edu//) as described previously.[14] Statistical Analysis of Microarrays [17] (SAM) was used to determine significantly differential expressed genes. A gene was considered as significantly differential expressed if the False Discovery Rate (FDR) was equal to or less than 5%. Cluster analysis[18] was used to define clusters of co-coordinately changed genes after which the data was visualized using Treeview.

Microarray data in this paper are stored in the publicly accessible Stanford Microarray Database website http://smd.stanford.edu/which supports the MIAME guidelines. In addition, data is stored in the Gene Expression Omnibus (GSE10655). The National Center for Biotechnology Information (www.ncbi.nlm.nih.gov/) Genbank accession numbers for the genes and gene products discussed in this paper are listed in Table S1.

### Realtime PCR

RNA (0.5 µg) was reverse transcribed into cDNA using a Revertaid H-minus cDNA synthesis kit (MBI Fermentas, St. Leon-Rot, Germany) according to the manufacturers' instructions. Quantitative realtime PCR was performed using an ABI Prism 7900HT Sequence detection system (Applied Biosystems, Foster City, CA, USA) using SybrGreen (Applied Biosystems). Primers were designed using Primer Express software and guidelines (Applied Biosystems) and are listed in table 1. To calculate arbitrary values of mRNA levels and to correct for differences in primer efficiencies a standard curve was constructed. Expression levels of target genes were expressed relative to housekeeping gene *glyceraldehydes-3-phosphate dehydrogenase* (*GAPDH*).

**Table 1:** Primers used for quantitative realtime PCR

Genes	Genbank accession nr.	Sense primer	Antisense primer	Length PCR product (bp)
*MxA*	NM 002462	TTCAGCACCTGATGGCCTATC	GTACGTCTGGAGCATGAAGAACTG	92
*OAS1*	NM 016816	TGCGCTCAGCTTCGTACTGA	GGTGGAGAACTCGCCCTCTT	175
*STAT1*	NM 007315	TGCATCATGGGCTTCATCAGC	GAAGTCAGGTTCGCCTCCGTTC	156
*RSAD2*	NM 080657	GTGGTTCCAGAATTATGGTGAGTATTT	CCACGGCCAATAAGGACATT	90
*IRF7*	NM 004031	GCTCCCCACGCTATACCATCTAC	GCCAGGGTTCCAGCTTCAC	99
*ISG15*	NM 005101	TTTGCCAGTACAGGAGCTTGTG	GGGTGATCTGCGCCTTCA	151
*IFNß*	NM 002176	ACAGACTTACAGGTTACCTCCGAAAC	CTCCTAGCCTGTCCCTCTGGGACTGG	93

### *In vitro* study

Freshly isolated PBMCs were washed using PBS containing 1% fetal calf serum (FCS; BioWhittaker, Cambrex) and plated in 24-wells culture plates at a density of 2×10^6^ cells per ml per well. Cells were left unstimulated or activated with 10 Units recombinant IFNß (Abcam, Cambridge, UK) for 4 h after which RNA was isolated using the Rneasy Qiagen RNA isolation kit (Qiagen) according to the manufacturers' instructions. A DNAse (Qiagen) step was included to remove genomic DNA. Quantity and purity of the RNA was tested using the Nanodrop spectrophotometer (Nanodrop Technologies, Wilmington, Delaware USA)

### Statistical analysis

Correlation analyses were performed using Graphpad Prism 4 software. First, data was tested for normal distribution. For normally distributed data, a Pearson correlation was used. A Spearman rank correlation was calculated in case of nonparametric distribution of the data. Correlations were considered significant if p-values were less than 0.05.

## Results

### Pharmacogenomics of IFNß therapy in MS

In order to understand the pharmacological effects of IFNß therapy we analysed the peripheral blood gene expression profiles of 16 RRMS patients at baseline and one month after the start of therapy. Two class paired analysis using Significant Analysis of Microarrays (SAM) at a False Discovery Rate (FDR) of less than 5% between pre- and post-therapy data was applied to identify genes that significantly changed in expression after IFNß treatment. Surprisingly, only 3 genes, “*Interferon alpha-inducible protein 27*” (*IFI27*), “*Tripartite motif-containing 69*” (*TRIM69*) and “*Epithelial stromal interaction protein 1 (breast)*” (*EPSTI1*), showed a significant change.

**Figure 1:**
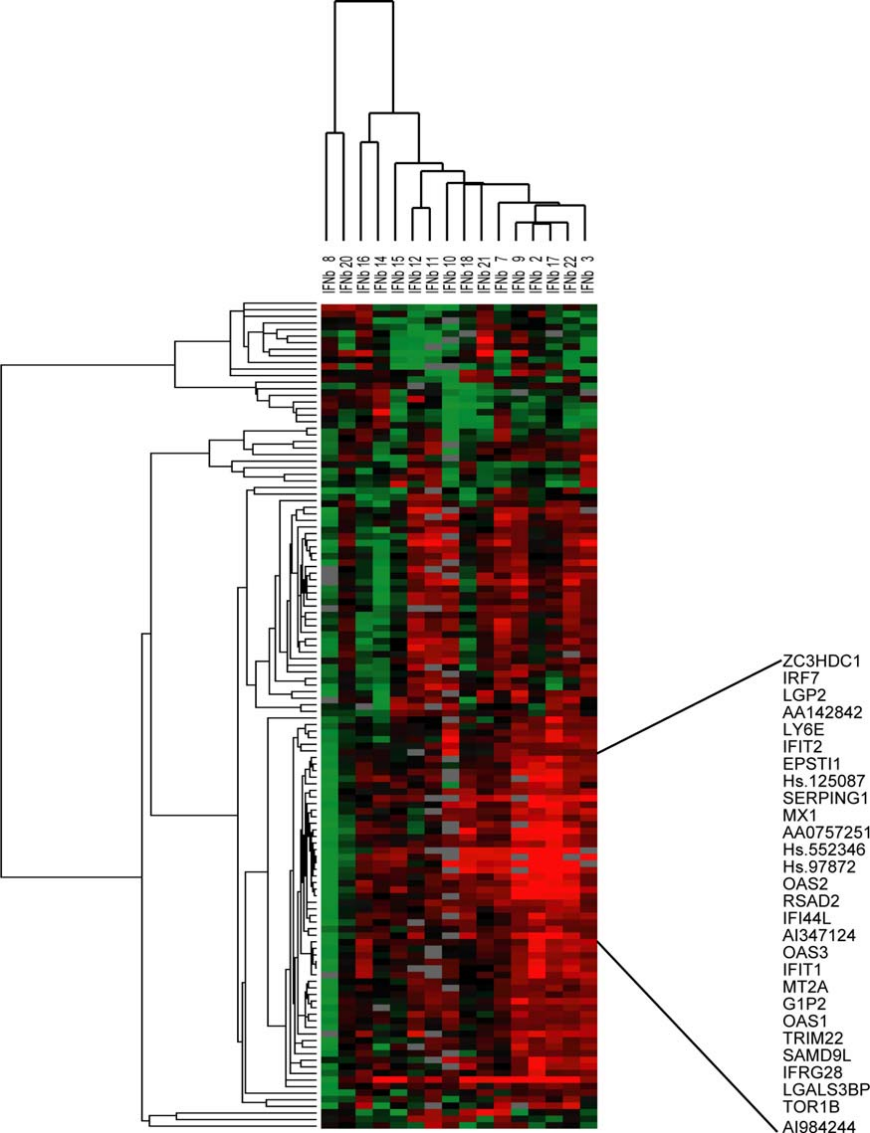
A. Biological response to IFNß therapy in MS patients Two-way hierarchical cluster analyses using gene expression ratio's (biological response). This diagram contains genes that were at least two-fold up- or downregulated after IFNß therapy in at least seven patients. Upregulated genes after therapy are indicated by a red colour, downregulated by a green colour and genes that show no differences in expression after therapy are indicated in black. B. Cluster of IFN-induced genes Selection of genes clustering together based on similar biological response profiles within the patient group. The genes clustered together with a correlation of 0.925 and are known to be induced by IFN. The mean expression ratio of all genes in this IFN cluster is referred to as the biological IFN response.

Given the heterogeneous nature of MS we questioned whether the observed poor yield of response genes upon IFNß treatment of the whole MS cohort could be a reflection of averaging out differences as a consequence of variation in pharmacological responsiveness between the patients. To test this hypothesis we investigated the pharmacological response at the individual patient level by calculating for each patient and for each gene the ratio of gene expression pre- *vs.* post therapy (log-2 ratios). We selected genes that showed at least a two-fold change in expression after IFNß treatment in at least 7 patients. A total of 126 genes met this criteria and were subsequently subjected to a two-way hierarchical (unsupervised) cluster analysis (Figure 1A). Compliant with our hypothesis, this analysis showed a marked variation in biological response to IFNß between patients. Some patients showed upregulated genes, whereas in other patients the same genes were downregulated or unchanged after IFNß therapy. As anticipated, part of this gene expression pattern is consistent with expression of known IFN response genes [13]. We next selected the cluster of genes showing the most inter-individual variation resulting in 28 IFN-induced genes (Table S1) that clustered tightly together (R = 0.925) indicating a coordinate regulation of these genes (Figure 1B). The expression data of some of the IFN-induced genes was validated by quantitative realtime PCR and showed a good correlation with the microarray data (Table 2). These findings confirmed the hypothesis that there exists considerable variation in the pharmacological effects of IFNß between patients with RRMS.

**Table 2:** Correlation between microarray data and realtime PCR data

Genes	p value	R value
*MxA*	0.0188	0.4335
*OAS1*	<0.0001	0.6972
*STAT1*	<0.0001	0.7371
*RSAD2*	<0.0001	0.7086
*IRF7*	0.0014	0.5648
*ISG15*	<0.0001	0.7051

### Relationship between pharmacological response and baseline gene expression levels

**Figure 2:**
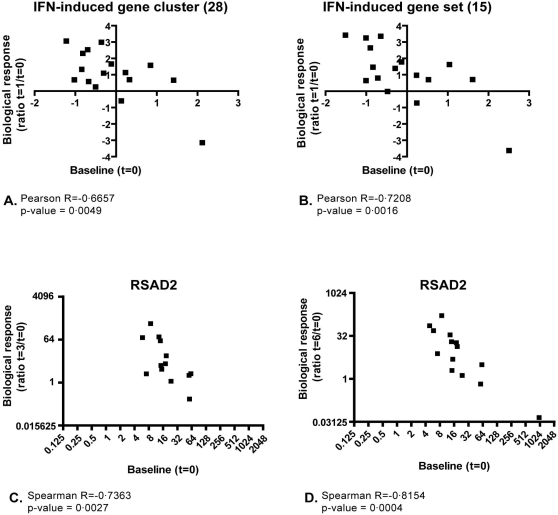
Correlation between baseline and biological response to IFNß therapy. Biological responses were calculated, using a set of IFN-induced genes (A and B) or a single IFN-induced gene (C and D) and correlated with baseline levels, resulting in a significant negative correlation. In C and D the expression levels of RSAD2 is measured by quantitative realtime PCR and normalized to the expression levels of *GAPDH*. A. IFN cluster as described in Figure 1B; B. Selection of 15 genes; C. Biological response after three months, using RSAD2 gene expression levels; D Biological response after six months using RSAD2 gene expression levels.

Previously, we demonstrated significant differences in the expression of type I IFN-induced genes between untreated RRMS patients.[14] Here we investigated whether there is a relationship between the differential *in vivo* responsiveness to IFNß and baseline expression levels of IFN-induced genes. Therefore, we tested for each patient whether there is an association between the mean expression levels of the IFN response gene cluster (shown in Figure 1B) before therapy with the response ratio after therapy. This analysis demonstrated that the mean baseline expression of the 28 IFN response genes negatively correlates with the *in vivo* IFN-induced response levels (p = 0.0049 and R = −0.6657) (Figure 2A), suggesting that the baseline gene expression level of these genes could serve a role as predictive marker for the pharmacological responsiveness to IFNß.

In order to create a gene set that best predicts the pharmacological response to IFNß we selected those genes whose expression shows the most significant negative correlation between baseline and biological response (with a cut off of p<0.01 and R<−0.65). This resulted in a gene set containing 15 genes (Table 3). Comparing baseline gene expression levels and biological response using the average of these 15 genes revealed a significant negative correlation (R = −0.7208; p = 0.0016) (figure 2B). To exclude a potential bias of the gene selection at baseline, we analyzed the correlation of the biological response determined by the mean expression value of the selected 15 IFN-induced genes with the baseline values of all genes on the array. This resulted in three additional genes (*IFI44L, MT1E and IMAGE:1879725*; R<−0.65 and variance >1.00) that significantly correlated with the pharmacological response to IFNß therapy. Although these genes did not cluster tightly together with the previously selected genes, they may be important in the response to IFNß.

**Table 3:** Correlation between baseline and therapy induced (ratio) expression levels measured at single gene level

Symbol	Accession number	p value	R value
*RSAD2*	NM_080657	0.0011	−0.7983
*IFIT1*	NM_001548	0.0004	−0.7746
*MX1*	NM_002462	0.0006	−0.7619
*ISG15*	NM_005101	0.0008	−0.7532
*IMAGE:1926927*	AI347124	0.0026	−0.7168
*EPSTI1*	NM_001002264	0.0059	−0.7162
*Transcribed locus*	Hs.552346	0.0038	−0.6977
*IRF7*	NM_004031	0.0029	−0.6925
*IMAGE:545138*	5′EST AA075776; 3′EST AA075725	0.0065	−0.6881
*LY6E*	NM_002346	0.0035	−0.6834
*OAS1*	NM_016816	0.0051	−0.6822
*OAS3*	NM_006187	0.0076	−0.6787
*IMAGE:504372*	AA142842	0.0087	−0.6707
*SERPING1*	NM_000062	0.0064	−0.6688
*Transcribed locus*	Hs.97872	0.0047	−0.6677
*IFI44L*	NM_006820	0.0196	−0.6353
*Transcribed locus*	Hs.125087	0.0108	−0.6175
*MT2A*	NM_005953	0.011	−0.6166
*TRIM22*	NM_006074	0.0118	−0.6115
*SAMD9L*	NM_152703	0.0121	−0.6102
*IMAGE:2562181 = OAS2*	NM_002535	0.0203	−0.591
*OAS2*	NM_002535	0.0169	−0.5865
*DHX58*	NM_024119	0.0222	−0.5842
*PARP12*	NM_022750	0.0343	−0.5482
*TOR1B*	NM_014506	0.0532	−0.4914
*IFIT2*	NM_001547	0.086	−0.4426
*RTP4*	NM_022147	0.1759	−0.369
*LGALS3BP*	NM_005567	0.314	−0.279

To investigate whether the observed negative correlation between baseline and treatment induced changes are stable over time we measure the expression level of the most significant correlating gene (RSAD2; see Table 3) again after three and six months of IFNß therapy. The negative correlation between baseline expression level and biological response was maintained after 3 months (p = 0.0027, R = −0.7363) and 6 months (p = 0.0004, R = −0.8154) of therapy (figure 2C and D). To validate our results, we measured expression levels of RSAD2 in a second independent group of patients (n = 30) before and after IFNß treatment. In this independent study group we confirmed the negative correlation between baseline gene expression level and treatment induced biological response (p<0.0085 and R = −0.4719).

### Comparative analyses of different treatment regimens

**Figure 3:**
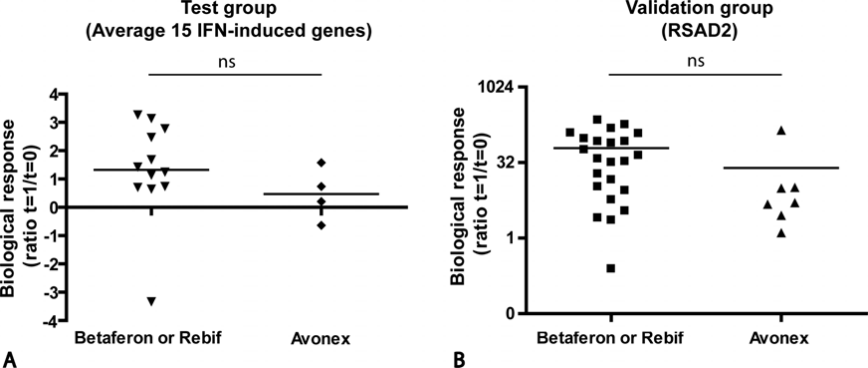
Comparative analysis between different treatment regimens. Comparison of biological response of Avonex treated patients and Betaferon or Rebif treated patients. A. Average biological response using the set of 15 IFN-induced genes in the test group of 16 RRMS patients; B. Biological response using PCR based gene expression levels for RSAD2 in the second independent validation group of 30 RRMS patients.

Since in the present study different pharmaceutical IFNβ preparations were used for treatment, we wanted to exclude the possibility of potential differences in pharmacokinetics and exposure as an explanation for our findings. Different studies have indicated no or negligible differences in bioavailability between different treatment preparations and routes of administration [19], [20]. To exclude a possible bias in our results due to differences in frequency of injection [20], [21] we divided our patients in two groups based on frequency of injection and compared their biological responses. One group of patients (group A) consists of patients with weekly treatment (Avonex) and the other group of patients (group B) who are treated three to four times a week (Rebif and Betaferon). Comparison of the response rates between the treatment groups revealed a similar range of response levels independent of the treatment regimen for both the test cohort (group A, n = 4 and group B, n = 12) based on microarray data, and the validation cohort (group A n = 6 and group B n = 24) based on quantitative PCR data (Figure 3). To provide further evidence that our results were not influenced by the frequency of injection we confirmed the negative correlation between the response rate and baseline IFN response gene expression in the group of frequently dosed patients (group B: test cohort (n = 12), R = −0.8361, p = 0.0007; validation cohort (n = 24), R = −0.4513, p = 0.0269).

Altogether, these results reveal that the observed negative correlation between baseline IFN signature and the extent of the biological response is not biased by the treatment regimen.

### Confirmation of ex vivo findings by in vitro IFNß stimulation of PBMC isolated at baseline

To further confirm that the observed inter-individual pharmacological differences were a consequence of differential responsiveness of peripheral blood cells and to exclude i. blood sampling error differences because of possible differential time-intervals between blood sampling and injection of IFNß, and ii. interference of inhibitory plasma proteins such as neutralizing antibodies, we performed an *in vitro* cell stimulation assay. Therefore we used purified PBMCs isolated prior to treatment, which were cultured for 4 hours in the presence of recombinant IFNß. To analyze the *in vitro* response to IFNß at baseline we measured the expression of a selected set of three known IFNß response genes and IFNß itself in resting and IFNß treated PBMCs by quantitative realtime PCR. The selected IFNß response genes were i. *RSAD2*, which showed the most significant correlation of biological response versus baseline at single gene level (Table 3), ii. *MxA*, which showed a good negative correlation and is known as a marker of IFN bioactivity, [22] and iii. *STAT1*, which is one of the components important for IFNß signaling. We hypothesized that baseline expression level of these genes influences subsequent IFNß signaling upon treatment. We compared the *in vitro* biological response of these genes to the mean *in vivo* biological response of the selected 15 genes. For all genes a significant correlation was revealed between the *in vitro* and *in vivo* biological response (Table 4). From these results we concluded that the differential IFNß responsiveness in MS is a consequence of intrinsic differences of peripheral blood cells in their responsiveness to IFNß. Moreover, the consistency between the *in vivo* and *in vitro* response to IFNß provides further evidence to exclude the involvement of different types and dosages of treatment on the observed pharmacological differences.

**Table 4:** Correlation between biological responses of single IFN-induced genes measured *in vitro* and mean biological response (using 15 genes) measured *in vivo*

Genes	p value	R value
*RSAD2*	0.0012	0.7518
*MxA*	0.0280	0.6064
*STAT1*	0.0100	0.6614
*IFNß*	0.0036	0.7675

### Biological IFN response and clinical parameters

The results described above could point towards a method to predict responsiveness to IFNß therapy based on baseline expression levels of IFN-induced genes. In the clinic the response status of a patient is measured by evaluation of Expanded Disability Status Scale (EDSS) progression, relapse rate and disease activity on Magnetic Resonance Imaging (MRI). For the first patient group (n = 16) EDSS progression, number of steroid interventions and relapse rate two years before initiation of treatment were assessed retrospectively and compared to the first two years after start of treatment. With this limited set of response criteria no association with the predictive pharmacological gene set of 15 IFN induced genes could be observed.

## Discussion

Our results reveal that RRMS patients show a heterogeneous pharmacological response to IFNß therapy. In some patients we demonstrate that administered exogenous IFNß induces functional activation of the IFN pathway, whereas other patients do not reveal a functional IFNß response. The latter are characterized by a biomarker profile reflecting a saturated IFN activation pathway prior to treatment. Hence the baseline expression of the biomarker profile reflecting the baseline status of the IFN activity negatively correlates with the pharmacological effects of IFNß treatment. This indicates that the baseline expression levels of the selected set of 15 IFN-induced genes can be used as a predictive marker for the responsiveness to IFNß treatment.

Thus patients with clinically defined similar disease may have intrinsic different modes of immune status. These findings make more evident the complexity of the disease and the relationship to therapy responsiveness.

Although different regimens of IFNß treatment were used in this study evidence is available that this does not affect our conclusions.

Firstly, there is accumulating evidence that there is no or little difference between different types of IFNβ in terms of their biological activity and routes of administration [19], [20]. Extent and duration of clinical and biologic effects were independent of the route of administration of IFNβ. Rebif when given *s.c.* or *i.m.* was found to be bioequivalent to Avonex [23], [24]. Moreover, there were no major differences between the results with IFNβ1a and 1b in the duration of the changes in the pharmacodynamic markers after the two routes of injection [25], [26].

Secondly, we excluded a possible bias in our results due to frequency of injection by analyzing different treatment groups separately. No significant differences in the range of biological response levels between Avonex treated patients and Rebif or Betaferon treated patients were observed, and selection of the high-frequently (Rebif and Betaferon) dosed patients by excluding weekly–treated (Avonex) patients from our analyses still resulted in a negative correlation between baseline IFN levels and biological response rate.

Thirdly, in the present study we show that the observed negative correlation between biological response and baseline levels of IFN induced genes is consistently observed over time, at one, three and six months after start of the therapy.

Finally, we showed that response-rates of *in vitro* stimulated PBMC isolated prior to treatment are consistent with those of the *ex vivo* results. These results convincingly supported the conclusion that the *in vivo* biological response is independent of differences in treatment regimens and interfering serum proteins such as neutralizing antibodies (Nabs).

Hence, we concluded that the inter-individual variation in pharmacological response to IFNß therapy is an intrinsic property of the peripheral blood cell compartment.

Several investigators have recently reported on transcription based responses to IFNß in MS. Baranzini and colleagues [27] used a pre-selected set of 70 genes and reported that (un)supervised two-way hierarchical clustering does not reveal significantly differential expressed genes between responders and non-responders. Using quadratic discriminant analysis-based integrated Bayesian inference system they found a gene triplet consisting of apoptosis-related genes as best predictive for good responder versus poor responder classification. Most of the 70 genes they selected are represented on our microarray but we didn't observe a difference for these genes using a gene-by-gene approach. However, the majority of genes that we found as predictive for responsiveness using an open survey approach were not present in the gene set selected by Baranzini and colleagues and therefore not identified in their study. A careful comparison between the different IFNβ pharmacogenomics studies [28], [29] learns that there is consistency between these reports and our data with respect to the heterogeneity of the IFNβ response. Although not explicitly mentioned in these reports, we learned that they contained evidence for inter-individual differences in response to IFNβ. Overall, despite basic differences in the designs, we confirm and extend the trends observed in these reports with respect to the heterogeneity in treatment response rates. In addition, our paired analysis method provides an ideal approach for a patient centric mode of data analysis and discloses significant differences in the expression of an IFN driven response gene set at baseline in relation to the pharmacological response. Our findings provide a perfect explanation for the inter-individual variation in the pharmacological responses mentioned above.

Our data based on paired analysis at the individual patient level clearly show that there is evidence for differences in IFNß responsiveness between patients with MS. The inter-individual differences in IFNß responsiveness may be the result of genetic variation in the IFNß biology.[2], [30] Feng and colleagues [31] showed that IFN-induced levels of mRNA and protein for IFN-regulatory genes (*IRF-1* and *IRF-2*) and antiviral genes (*MxA* and *2′, 5′-OAS*) were significantly lower in PBMC from patients with clinically active MS compared to normal controls. They demonstrated that clinical disease activity was related to decreased phosphorylation of Ser-STAT-1 and proposed that this could be a mechanism explaining a defective IFN response. Whereas these studies provided insight into the IFN responsiveness in terms of a group average the issue of inter-individual heterogeneity was not addressed. Other mechanisms that could account for differential responsiveness to IFNß include variation in activity of inhibitory transcription factors. Evidence exists that crosstalk with other cytokine-activated pathways, could cause tachyphylaxis to type I IFNs. Although type I IFNs have an essential function in mediating innate immune responses against viruses, they may already be produced at very low levels in the absence of viral infections [32] in serum of a subset of MS patients. Since e.g. IFNα is known to desensitize further responses to IFNs, the presence of low endogenous IFNs could block IFNß-induced signals.[33], [34]

This explorative pilot study suggests a predictive value of baseline gene expression levels of IFN-induced genes. Since the molecular differences most likely reflect distinct pathophysiologic processes underlying disease, it is tempting to speculate that these differences will predict individual responsiveness to treatment. Clinical response to IFNß may be determined by disability progression and relapse rate. Because MS is a chronic disease with an unpredictable clinical course it remains difficult to assess clinical responder status at an individual patient level. A more objective method for determining disease activity is the measurement of MRI parameters, e.g. CNS atrophy measures or T1 gadolinium enhancing or the appearance of new T2 lesions.[3], [35], [36] However, using these methods it is still extremely difficult to precisely define the state of responsiveness after a short period of treatment or preferably before start of the treatment. These facts emphasize the importance of finding pharmacological predictors and/or determinants for treatment responsiveness. We realize that the design of this study does not allow any firm conclusions to be drawn concerning the clinical parameters associated with the molecular phenotype.

Hence, further studies in a large cohort of patients starting IFNß treatment are needed to validate and further investigate the predictive value of baseline IFN response gene expression levels and it is of great importance to find a correlation between clinical parameters and the biological IFN response. In future, molecular stratification of patients at baseline may be helpful in assembling homogeneous populations of patients, which will improve the likelihood of observing drug efficacy in clinical trials.

## Supporting Information

Table S1 Gene details for the cluster of 28 genes shown in figure 1B
(0.05 MB DOC)
