# Supplementary material for: Pharmacogenomics of Interferon-ß Therapy in Multiple Sclerosis: Baseline IFN Signature Determines Pharmacological Differences between Patients
Source: PLoS One. 2008 Apr 2;3(4):e1927. doi: 10.1371/journal.pone.0001927 (PMC2271130; doi:10.1371/journal.pone.0001927)
Supplement: Table S1 — Gene details for the cluster of 28 genes shown in figure 1B (0.05 MB DOC) [file pone.0001927.s001.doc]

| **Symbol** | **Name** | **Accession number GenBank** |
| --- | --- | --- |
| DHX58 | DEXH (Asp-Glu-X-His) box polypeptide 58 | NM_024119 |
| EPSTI1 | Epithelial stromal interaction 1 (breast) | NM_001002264 |
| IFI27 | Interferon, alpha-inducible protein 27 | NM_005532 |
| IFI44L | Interferon-induced protein 44-like | NM_006820 |
| IFIT1 | Interferon-induced protein with tetratricopeptide repeats 1 | NM_001548 |
| IFIT2 | Interferon-induced protein with tetratricopeptide repeats 2 | NM_001547 |
| IMAGE:1926927 |  | AI347124 |
| IMAGE:2562181 | 2'-5'-oligoadenylate synthetase 2, 69/71kDa | NM_002535 |
| IMAGE:504372 |  | AA142842 |
| IMAGE:545138 |  | 5'EST AA075776; 3'EST AA075725 |
| IRF7 | Interferon regulatory factor 7 | NM_004031 |
| ISG15 | ISG15 ubiquitin-like modifier | NM_005101 |
| LGALS3BP | Lectin, galactoside-binding, soluble, 3 binding protein | NM_005567 |
| LY6E | Lymphocyte antigen 6 complex, locus E | NM_002346 |
| MT1E | Metallothionein 1E | NM_175617 |
| MT2A | Metallothionein 2A | NM_005953 |
| MX1 | Myxovirus (influenza virus) resistance 1, interferon-inducible protein p78 (mouse) | NM_002462 |
| OAS1 | 2',5'-oligoadenylate synthetase 1, 40/46kDa | NM_016816 |
| OAS2 | 2'-5'-oligoadenylate synthetase 2, 69/71kDa | NM_002535 |
| OAS3 | 2'-5'-oligoadenylate synthetase 3, 100kDa | NM_006187 |
| PARP12 | Poly (ADP-ribose) polymerase family, member 12 | NM_022750 |
| RSAD2 | Radical S-adenosyl methionine domain containing 2 | NM_080657 |
| RTP4 | Receptor (chemosensory) transporter protein 4 | NM_022147 |
| SAMD9L | Sterile alpha motif domain containing 9-like | NM_152703 |
| SERPING1 | Serpin peptidase inhibitor, clade G (C1 inhibitor), member 1, (angioedema, hereditary) | NM_000062 |
| TOR1B | Torsin family 1, member B (torsin B) | NM_014506 |
| TRIM22 | tripartite motif-containing 22 | NM_006074 |
| TRIM69 | Tripartite motif-containing 69 | NM_182985 |
